# Scl-Ab reverts pro-osteoclastogenic signalling and resorption in estrogen deficient osteocytes

**DOI:** 10.1186/s12860-020-00322-w

**Published:** 2020-11-04

**Authors:** H. Allison, G. Holdsworth, L. M. McNamara

**Affiliations:** 1 Mechanobiology and Medical Devices Research Group (MMDRG), Centre for Biomechanics Research (BioMEC), Biomedical Engineering, College of Engineering and Informatics, National University of Ireland Galway, Galway, Ireland; 2 Early Solutions, UCB Pharma, Slough, UK

**Keywords:** Mechanobiology, Estrogen deficiency, Sclerostin antibody, Osteoclastogenesis, Oscillatory fluid flow

## Abstract

**Background:**

Neutralising antibodies to sclerostin (Scl-Ab) have shown significant potential to induce bone formation and decrease bone resorption, increase strength and substantially reduce fracture risk in animal studies and clinical trials. Mechanical loading negatively regulates sclerostin expression, and sclerostin has been shown to induce RANKL synthesis in osteocytes. However, how Scl-Ab governs osteocyte regulation of osteoclast differentiation and function is not fully understood. We have recently discovered that osteoblasts and osteocytes alter osteoclastogenic signalling (RANKL/OPG) during estrogen-deficiency, and that osteoblast-induced osteoclastogenesis and resorption are exacerbated. However, it is not known whether estrogen deficient osteocytes exacerbate osteoclastogenesis. The aims of this study were to (1) establish whether osteocytes induce osteoclastogenesis and bone resorption during estrogen deficiency in vitro (2) investigate whether the sclerostin antibody can revert osteocyte-mediated osteoclastogenesis and resorption by attenuating *RANKL*/*OPG* expression.

**Results:**

Using conditioned media and co-culture experiments we found increased osteocyte-induced osteoclastogenesis and bone resorption in estrogen deficient conditions. This is the first study to report that administration of Scl-Ab has the ability to revert osteocyte-mediated osteoclastogenesis and resorption by decreasing *RANKL*/*OPG* ratio expression and increasing *WISP1* expression in estrogen deficient osteocytes.

**Conclusions:**

This study provides an enhanced understanding of the biological changes underpinning decreases in bone resorption following Scl-Ab treatment observed in vivo by revealing that Scl-Ab can reduce pro-osteoclastogenic cell signalling between osteocytes and osteoclasts.

## Background

Bone loss during postmenopausal osteoporosis is primarily attributed to osteoclast resorption [1], and for this reason anti-resorptive therapies, such as bisphosphonates, are widely used to inhibit osteoclast function and prevent bone loss [2]. However, anti-resorptive therapies only reduce fracture susceptibility by up to 50% [3]. Anabolic therapies have emerged, which promote new bone formation by targeting osteoblast and osteocyte activity [4–6]. In particular, neutralising antibodies to sclerostin (Scl-Ab) have shown significant potential to induce new bone formation, increase bone mass and strength, and substantially reduce fracture risk in animal studies and clinical trials [4, 7–11]. Moreover, the Scl-Ab also decreases bone resorption in an indirect fashion [4, 9, 12], yet the cellular changes underpinning this effect are not fully understood.

Osteocytes are the primary mechanosensors in bone, and it is thought that they are able to detect mechanical stimuli using mechanosensitive proteins, such as primary cilia, gap junctions and integrins [13–15], and transduce mechanical stimuli into biochemical responses [16, 17]. Osteocytes regulate bone remodelling by signalling to both osteoblasts and osteoclasts via soluble paracrine factors, and direct cell-cell contact [18]. RANKL is a cytokine produced by osteocytes, osteoblasts and stromal cells, which induces osteoclastogenesis in cells of the monocyte/macrophage lineage [19–21]. OPG is also produced by osteocytes, osteoblasts and stromal cells, and acts as a decoy receptor for RANKL and thereby inhibits osteoclastogenesis [22]. Thus, the ratio of RANKL and OPG in bone is a major determinant of bone mass and strength [23, 24]. In vitro studies have demonstrated that mechanically stimulating MLO-Y4 cells leads to decreases in *RANKL*/*OPG* ratio and a reduction in osteoclastogenesis when co-cultured with RAW264.7 and BMM cells [25–27]. Estrogen plays a role in osteocyte mechanosensation; in vitro studies have demonstrated that estrogen treated MLO-Y4 cells exhibited increased NOS activity, NO and PGE_2_ release, as well as increased intracellular calcium [Ca^2+^] oscillations in response to fluid flow [28]. Interestingly, biochemicals NO and PGE_2_ are known to promote bone formation and inhibit osteoclast activity [29–31]. However, when estrogen was withdrawn from MLO-Y4 cells or the estrogen receptor chemically inhibited, intracellular [Ca^2+^] calcium oscillations and the downstream responses to fluid flow were reduced [28]. Moreover, the putative osteocyte integrin α_υ_β_3_ mechanosensor is affected in estrogen deficient conditions both in vivo and in vitro. In vivo the number of β_3_ integrin-positive osteocytes was reduced in cortical bone of ovariectomised (OVX) rats compared to SHAM animals [32]. In vitro estrogen deficient MLO-Y4 cells have been shown to have smaller focal adhesion area with reduced α_υ_β_3_ localisation [27]. Furthermore, the estrogen deficient MLO-Y4 cells displayed an increase in *RANKL*/*OPG* ratio as well as defective *COX-2* expression in response to fluid flow in a similar manner to MLO-Y4 cells cultured under conditions that inhibited the α_υ_β_3_ integrin [27]. Although such findings suggest that osteocytes regulation of osteoclasts should be disrupted, the direct effect of altered paracrine signalling from estrogen deficient osteocytes on osteoclastogenesis and osteoclast resorption has never been investigated.

The Wnt antagonist sclerostin (encoded by the *SOST* gene), produced by mature osteocytes, binds to LRP5/6 Wnt co-receptors, negatively regulates osteoblast proliferation and differentiation via inhibition of the Wnt/β-catenin signalling pathway, and also promotes osteocyte and osteoblast apoptosis [33]. Following mechanical loading, *SOST* mRNA and sclerostin protein expression are downregulated both in vivo [34] and in vitro in the osteocyte cell line OCY454 [35]. Estrogen has been observed to negatively affect *SOST* mRNA and sclerostin protein expression in human postmenopausal bone and ovariectomised mice respectively [36, 37]. In contrast to estrogen negatively regulating *SOST* expression, one study found that *SOST* expression was reduced in estrogen deficient mice [38]. Thus, the effect of estrogen on *SOST* expression in vitro is not yet fully understood. There are other known antagonists of the Wnt signalling pathway such as Wnt inhibitory factor 1 (*WIF1*), Frizzled-related protein (*FRZB*) and Secreted frizzled related protein 2 (*SFRP2*) [39, 40]. *SFRP2* is rapidly downregulated in uterine stromal cells from wild-type (WT) and estrogen receptor α deficient (ER-α −/−) when treated with the estrogen receptor antagonist Fulvestrant [41]. However it is not known whether estrogen also affects expression of other Wnt antagonists in bone cells. Although it has been reported that sclerostin increases *RANKL* expression in MLO-Y4 cells [42], and Scl-Ab treatment is effective for increasing bone formation and reducing bone resorption in OVX animals and postmenopausal women [4, 8–10, 12], how Scl-Ab governs osteocyte regulation of osteoclast differentiation and function is not yet fully understood. Transcriptional profiling of laser capture microdissected osteocytes in bone from rats treated with a single dose of 100 mg/kg Scl-Ab revealed early expression changes in regulators of osteoclastogenesis [43]. Specifically, *DLX5* (a positive regulator of osteoblastogenesis) was upregulated 72 and 168 h after receiving the Scl-Ab, *WISP1* (a negative regulator of osteoclastogenesis) was also significantly and consistently upregulated following Scl-Ab administration. However, *CXCL14* and *CXCL12* were downregulated 24 and 168 h respectively after receiving Scl-Ab [43]. *DLX5* knockout mice have displayed increases in osteoclast formation and increased *RANKL*/*OPG* expression in osteoblasts [44]. *WISP* knockout mice revealed that *WISP1* is negative regulator of osteoclastogenesis and stimulates osteoblasts [45]. *CXCL14* is a chemoattractant chemokine for macrophages [46] and *CXCL12* binding to its receptor *CXCR4* has be shown to promote chemotactic recruitment, development and survival of osteoclasts [47]. In vitro studies have not yet been conducted to understand the biological mechanisms behind sclerostin inhibition and its role in reducing osteocyte-induced osteoclastogenesis and resorption during estrogen deficiency.

In this study the hypotheses that (1) mechanically stimulated osteocytes induce osteoclastogenesis and bone resorption during estrogen deficiency and (2) inhibiting sclerostin reduces osteocyte-induced osteoclastogenesis in vitro, were tested. These studies implement mechanobiology experiments on osteocytes, and their conditioned medium, and osteocytes with BMM or RAW264.7 cells in co-culture to investigate (1) in vitro osteocyte-induced osteoclastogenesis and resorption following loading and estrogen deficiency, (2) changes in osteocyte gene expression of Wnt antagonist’s in estrogen and estrogen deficient conditions and (3) whether Scl-Ab administration reverts pro-osteoclastogenic signalling in estrogen deficient osteocytes.

## Results

### Estrogen deficiency promotes osteocyte production of soluble pro-osteoclastogenic factors resulting in increased bone resorption. Inhibiting sclerostin can reduce osteoclastogenesis and resorption

**Fig. 1:**
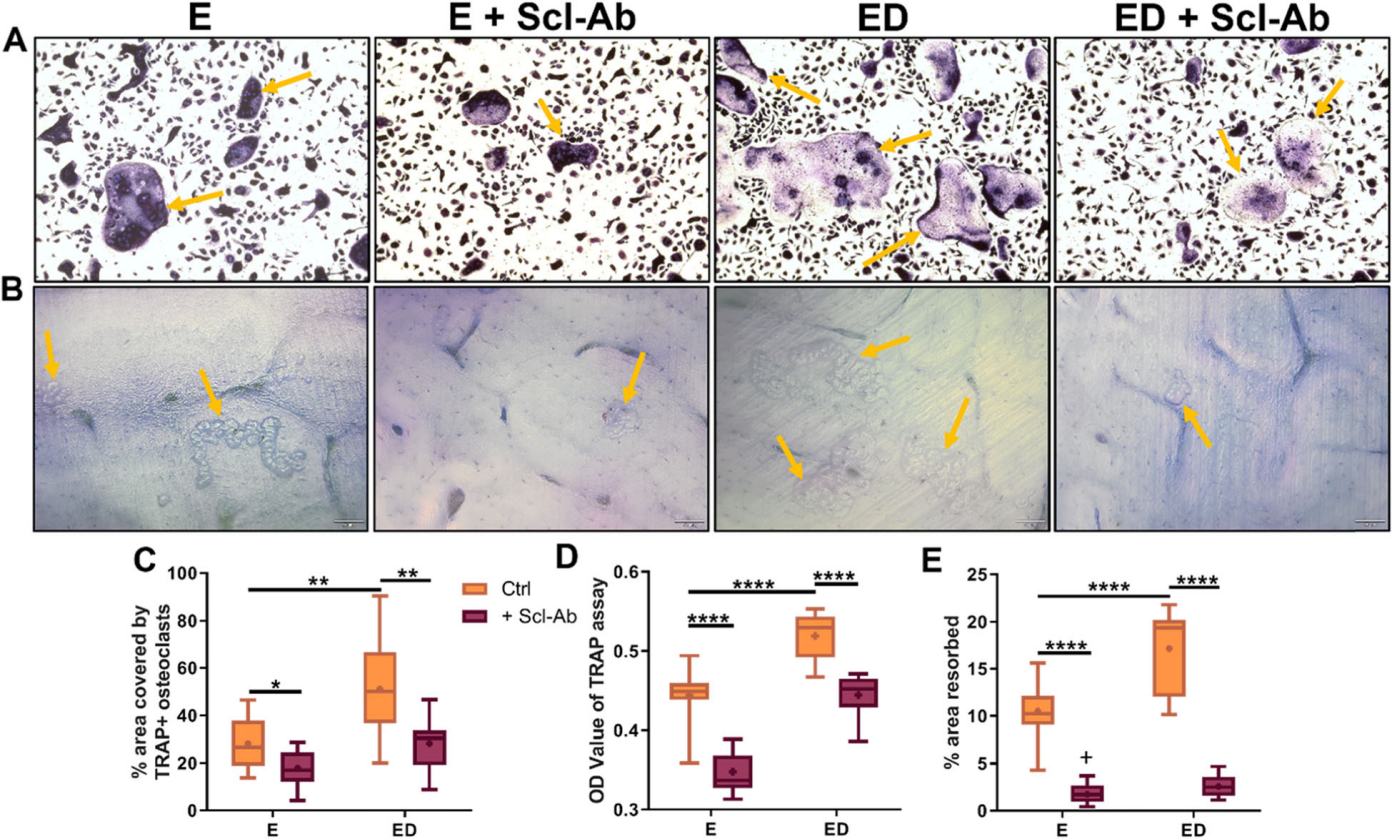
The effect of CM from OCY454 cells on osteoclastogenesis and bone resorption. Images show (**a**) multinucleated TRAP+ osteoclasts (orange arrows) (*N* = 3, *n* = 9) and **b** bone resorption pits and trails (orange arrows) (*N* = 2, *n* = 8) after BMM cells were treated with CM from OCY454 cells. Quantification of images showing (**c**) percentage surface area covered by TRAP+ multinucleated cells (*N* = 3, *n* = 9), **d** TRAP activity in supernatant collected from multinucleated cells (*N* = 3, *n* = 9) and **e** percentage area resorbed by osteoclasts (*N* = 2, *n* = 8) (‘+’ indicates a significant interaction (*p* < 0.001) between the inhibitory effects of estrogen and Scl-Ab on bone resorption as determined by two-way ANOVA). E, estrogen treatment; ED, Estrogen deficient; Scl-Ab, Sclerostin antibody. Student’s t-test. * = *p* < 0.05, ** = *p* < 0.01 and **** = *p* < 0.0001

In vitro*,* osteocytes that have undergone an estrogen withdrawal regime have been shown to have impaired mechanosensation and altered pro-osteoclastogenic mRNA expression (*RANKL*/*OPG*, *COX-2*) [27]. To mimic the conditions observed in estrogen deficiency, osteocytes were subjected to an estrogen withdrawal regime, in which estrogen was withdrawn from the cells that had been accustomed to estrogen for 3 days. Following oscillatory fluid flow, CM was collected and CM experiments were conducted to investigate the paracrine signalling between mechanically stimulated osteocytes and osteoclast precursors. There was significantly higher TRAP+ cells when BMMs were cultured with CM from estrogen deficient (ED) osteocytes (*p* < 0.01) and also higher levels of TRAP activity (*p* < 0.0001) compared to CM from estrogen treated osteocytes (E) (Fig. 1a, c, d). Bovine bone discs were used to assess the resorptive ability of the differentiated osteoclasts and a significant increase in resorption was observed when BMM cells were cultured with CM from estrogen deficient osteocytes compared to CM from estrogen treated osteocytes (*p* < 0.0001) (Fig. 1b, e). An increase in osteoclastogenesis and TRAP activity was also observed when RAW264.7 cells were treated with CM from estrogen deficient osteocytes compared to estrogen treated osteocytes (Supplementary Figure 1).

Scl-Ab administration in vivo has shown to prevent bone loss induced by mechanical unloading and ovariectomy in a rat model of osteoporosis [4]. Here we examined whether administration of Scl-Ab to estrogen deficient osteocytes in vitro could reduce the increase in osteoclastogenesis observed when BMMs were exposed to CM from estrogen deficient osteocytes. When estrogen-treated osteocytes were treated with Scl-Ab, they produced a CM that decreased osteoclastogenesis (*p* < 0.05) and TRAP activity (*p* < 0.0001) in BMMs further, when compared to estrogen-treated cells that received no Scl-Ab (Fig. 1a, c, d). Similarly, there was a significant decrease in osteoclastogenesis (*p* < 0.01) and TRAP activity (*p* < 0.0001) when BMMs were treated with CM from estrogen deficient osteocytes treated with the Scl-Ab when compared to CM from untreated estrogen deficient osteocytes (Fig. 1a, c, d). Bone resorption by BMM was significantly reduced (*p* < 0.0001) when receiving CM from osteocytes exposed to Scl-Ab, when compared to groups receiving CM from estrogen-treated or estrogen deficient osteocytes with no antibody (Fig. 1b, e). In addition, a two-way ANOVA revealed a significant interaction between the inhibitory effects of estrogen and Scl-Ab on bone resorption (*p* = 0.0002; Fig. 1e). However, analysis by two-way ANOVA revealed no significant interaction between the effects of estrogen and Scl-Ab on osteoclastogenesis or TRAP activity. Increased osteoclastogenesis and TRAP activity was also observed when RAW264.7 cells were treated with CM from estrogen deficient osteocytes compared to estrogen treated osteocytes. However, CM collected from osteocytes exposed to Scl-Ab did not have the same inhibitory effect on RAW264.7, which was observed in BMM cell cultures (Supplementary Figure 1).

In summary, estrogen deficient and mechanically loaded osteocytes produce soluble factors that increase osteoclastogenesis, TRAP activity and bone resorption when compared to estrogen treated and mechanically loaded osteocytes. However, inhibiting sclerostin reduces pro-osteoclastogenic paracrine signalling between osteocytes and osteoclast pre-cursors (BMM cells) under both estrogen and estrogen deficient conditions.

### Estrogen deficient osteocytes treated with Scl-Ab produce soluble factors which downregulate CTSK and NFATc1 expression in osteoclasts

**Fig. 2:**
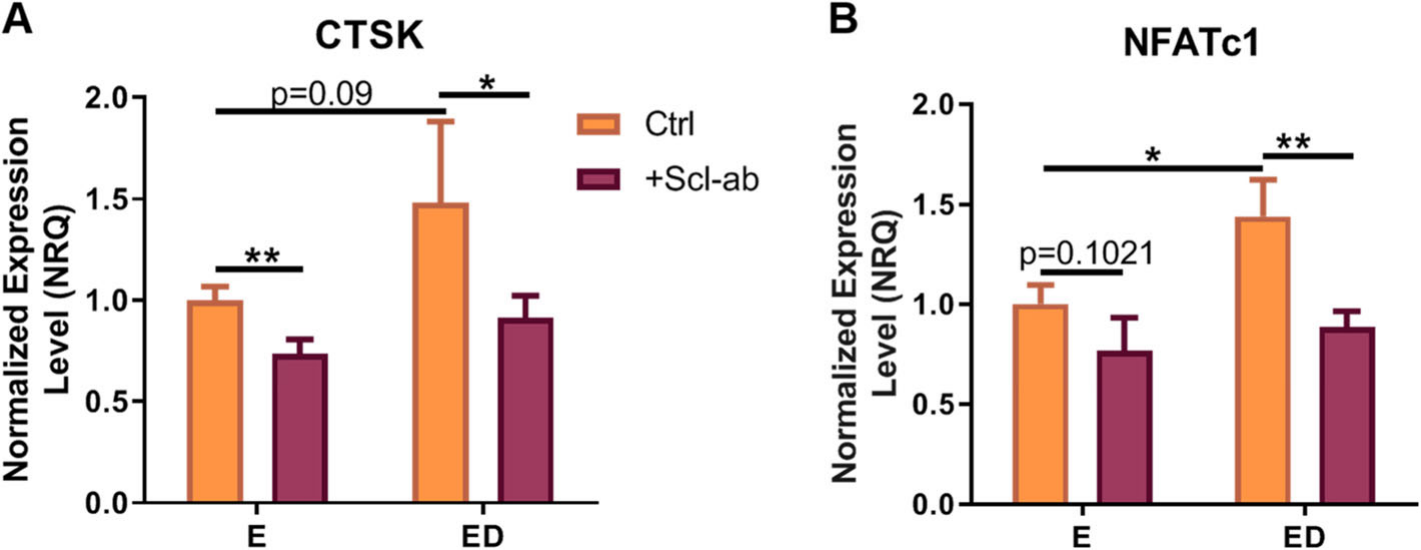
The effect of CM from OCY454 cells on the expression of osteoclast genes in BMM cells. qRT-PCR analysis of (**a**) CTSK expression (*N* = 3, *n* = 9) and **b** NFATc1 expression (*N* = 3, *n* = 9). E, estrogen treatment; ED, Estrogen deficient; Scl-Ab, Sclerostin antibody. Student’s t-test.* = *p* < 0.05 and ** = *p* < 0.01

*NFATc1* is a master regulator of osteoclast differentiation, and regulates a number of osteoclast specific gene such as *TRAP* and *CTSK* [48]. *CTSK* transcript encodes for cathepsin K, a protease which breaks down type I collagen and therefore plays an important role in bone resorption [49]. We assessed the effects osteocytes CM had on both *CTSK* and *NFATc1* expression in BMM cells. BMM cells upregulated the expression of *NFATc1* (*p* < 0.05) following 5 days of culture with CM from estrogen deficient osteocytes compared to CM from estrogen-treated cells but not *CTSK* expression (*p* = 0.09) and (Fig. 2a, b). Supporting the downregulation in bone resorption observed, the CM from osteocytes exposed to Scl-Ab significantly downregulated BMM *CTSK* expression when compared to the relevant CM from either estrogen-treated or estrogen deficient osteocytes that received no antibody (*p* < 0.01 and *p* < 0.05 respectively) (Fig. 2a). No significant difference in *NFATc1* expression was observed when BMM cells were treated with CM from estrogen and Scl-Ab treated osteocytes compared to CM from osteocytes treated with estrogen only. However, a downregulation in *NFATc1* expression was observed when BMM cells were treated with CM from Scl-Ab treated estrogen deficient osteocytes compared to CM from untreated estrogen deficient osteocytes (*p* < 0.01) (Fig. 2b). Analysis by two-way ANOVA revealed no significant interaction between the effects of estrogen and Scl-Ab on *NFATc1* and *CTSK* expression. Taken together, estrogen deficient osteocytes treated with Scl-Ab produce soluble factors that downregulate the expression of genes necessary for normal osteoclast formation and function.

### Estrogen deficient osteocytes that were mechanically stimulated induce osteoclastogenesis in a direct cell-cell contact co-culture system, but in the presence of Scl-Ab osteoclastogenesis is attenuated

**Fig. 3:**
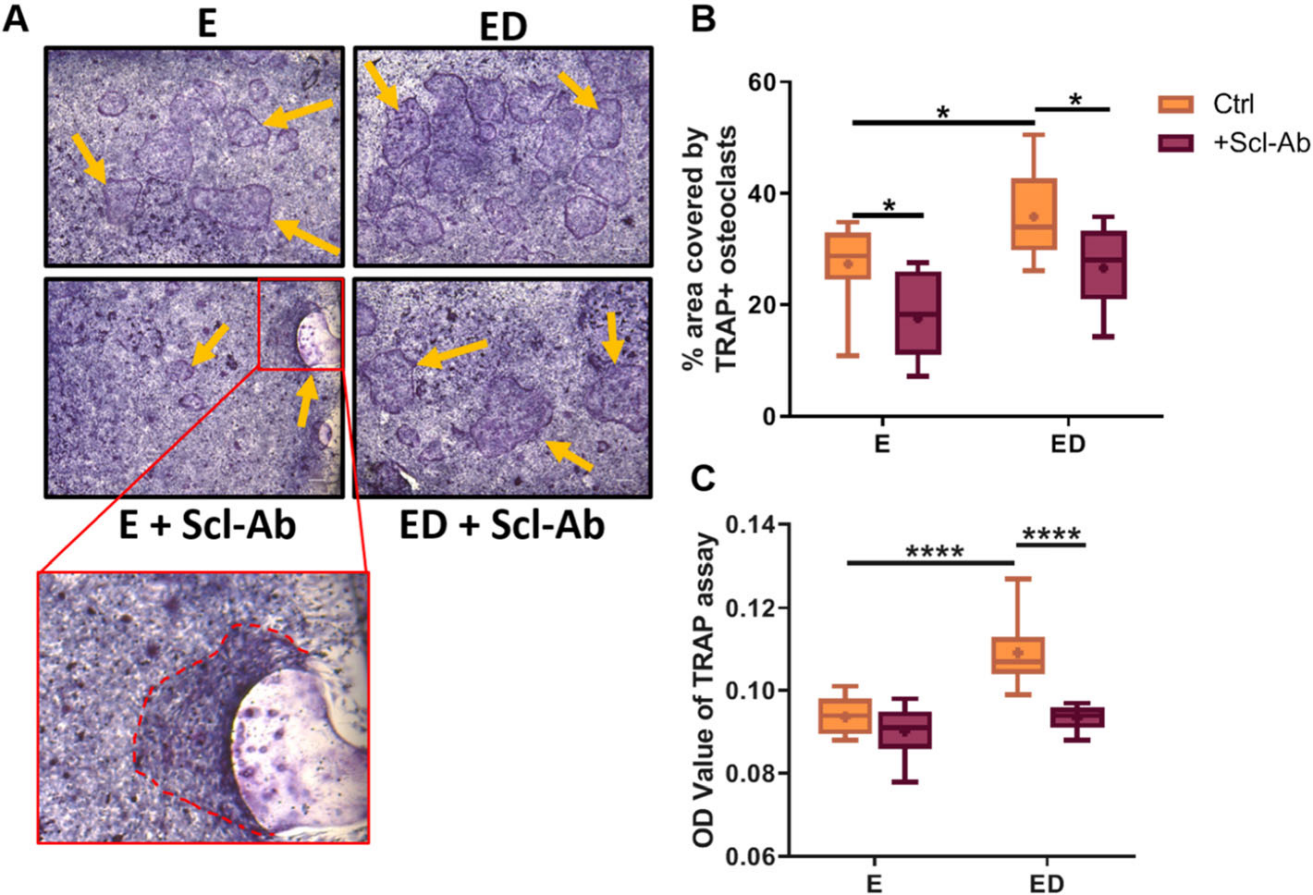
The effect of sclerostin inhibition on osteocyte induced osteoclastogenesis in a co-culture system. **a** Images show TRAP+ osteoclasts (orange arrows) formed when BMM cells were co-cultured with OCY454 cells for 6 days. (*N* = 3, *n* = 9). Quantification of images showing (**b**) percentage area covered by the TRAP+ cells (*N* = 3, *n* = 9) and **c** TRAP activity in supernatant collected from OCY454-BMM co-cultures after 6 days (*N* = 3, *n* = 9). E, estrogen treatment; ED, Estrogen deficient; Scl-Ab, Sclerostin antibody. Student’s t-test. * = *p* < 0.05 and **** = *p* < 0.0001

Cell-cell co-culture studies have previously revealed that mechanically stimulated MLO-Y4 cells inhibit osteoclastogenesis in both mouse BMMs and RAW264.7 cells [25, 26]. However, it is unknown what effect estrogen deficiency has on this process, and for this reason we conducted co-culture studies in which BMM cells were seeded on top of mechanically stimulated osteocytes that had previously received either estrogen treatment or an estrogen withdrawal regime. We report that estrogen deficient osteocytes had an enhanced capacity to induce osteoclast differentiation in BMM cells when compared to estrogen-treated cells (*p* < 0.05) and a concomitant increase in TRAP activity (*p* < 0.0001) was also seen (Fig. 3a, b, c). Co-cultures with RAW264.7 cells produced similar results (Supplementary Figure 2).

We then assessed the effects of inhibiting sclerostin on osteocyte-osteoclast cell-cell signalling. In estrogen treated osteocytes, Scl-Ab treatment decreased osteoclastogenesis in BMMs (*p* < 0.05) but had no significant effect on TRAP activity (Fig. 3b, c). Similar results were seen when estrogen treated osteocytes were co-cultured with RAW264.7 cells (Supplementary Figure 2). Administration of Scl-Ab reduced osteoclastogenesis (*p* < 0.05) and TRAP activity (*p* < 0.0001) when BMMs were co-cultured with estrogen deficient osteocytes and indeed these reverted to levels comparable with estrogen-treated co-cultures (Fig. 3b, c). Similar to the CM experiments, analysis by two-way ANOVA revealed no significant interaction between the effects of estrogen and Scl-Ab on osteoclastogenesis or TRAP activity in the co-culture systems.

In summary, mechanically stimulated estrogen deficient osteocytes have an enhanced capacity to induce osteoclastogenesis in a BMM co-culture system, when compared to estrogen-treated osteocytes which have also received mechanical stimulation. However, when estrogen deficient osteocytes are co-cultured with BMM cells in the presence of Scl-Ab, osteoclastogenesis is attenuated.

### RANKL/OPG ratio expression is increased in estrogen deficient osteocytes, but this increase is inhibited following Scl-Ab administration

**Fig. 4:**
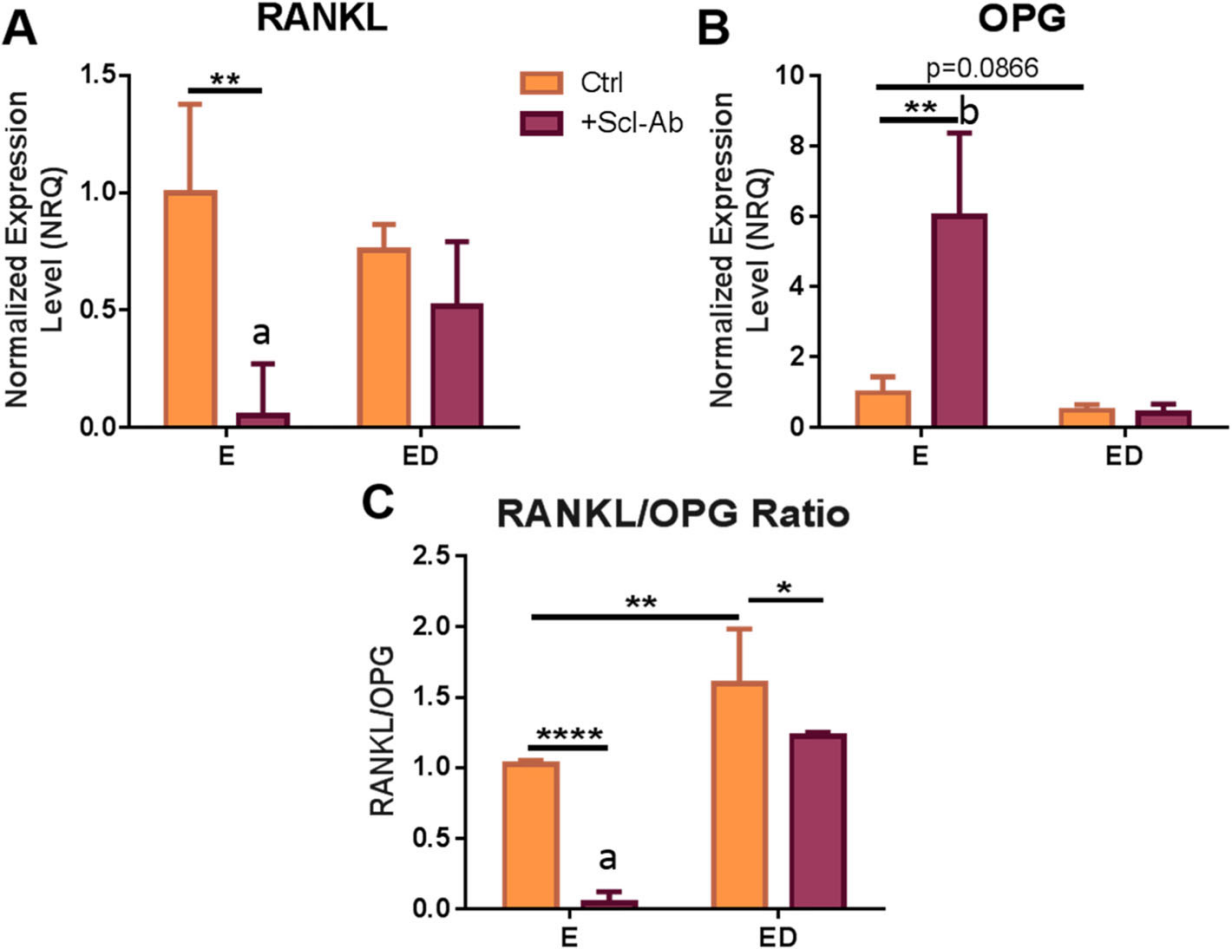
The effect of estrogen deficiency and sclerostin inhibition on RANKL and OPG gene expression. qRT-PCR analysis of gene expression (relative to estrogen treated group) in OCY454 cells 24 h after mechanical stimulation (**a**) RANKL expression (*N* = 3, *n* = 9), **b** OPG expression (*N* = 3, *n* = 9) and **c** RANKL to OPG ratio (*N* = 3, *n* = 9). (‘a’ and ‘b’ indicates a significant interaction (*p* < 0.05 and *p* < 0.01 respectively) between the effects of estrogen and Scl-Ab on RANKL and OPG expression as determined by two-way ANOVA) E, estrogen treatment; ED, Estrogen deficient; Scl-Ab, Sclerostin antibody. Student’s t-test. * = *p* < 0.05, ** = *p* < 0.01 and **** = *p* < 0.0001

Osteocytes are the main source of RANKL, a cytokine that induces osteoclastogenesis [50]. We assessed whether the increases in osteoclastogenesis when BMMs cells were co-cultured with estrogen deficient osteocytes, or it’s CM, could be explained by changes in *RANKL* or *OPG* expression levels. There was no statistically significant difference in *RANKL* or *OPG* expression levels between estrogen deficient and estrogen-treated osteocytes (Fig. 4a, b). However, the *RANKL*/*OPG* ratio increased significantly in estrogen deficient cells compared to estrogen treated osteocytes (*p* < 0.01) (Fig. 4c).

Scl-Ab altered the *RANKL*/*OPG* ratio in estrogen-treated osteocytes; there was a significant down-regulation in *RANKL* expression (*p* < 0.01) and an up-regulation in *OPG* expression (*p* < 0.01), resulting in a decrease in *RANKL*/*OPG* ratio (*p* < 0.0001) compared to estrogen-treated osteocytes that received no Scl-Ab (Fig. 4a, b, c). Analysis by two-way ANOVA revealed a significant interaction between the effect of estrogen and the effect of Scl-Ab administration on the expression of these genes (*RANKL*: *p* = 0.0194, *OPG*: *p* = 0.0012 and *RANKL*/*OPG*: *p* = 0.0118; Fig. 4). In estrogen deficient osteocytes, Scl-Ab did not alter *RANKL* or *OPG* expression individually (Fig. 4a, b). However, the *RANKL*/*OPG* ratio was decreased in estrogen deficient osteocytes treated with Scl-Ab compared estrogen deficient osteocytes who received no Scl-Ab treatment (*p* < 0.05) (Fig. 4c).

Taken together, these results demonstrate that estrogen withdrawal leads to an increase in *RANKL*/*OPG* ratio expression compared to estrogen treated osteocytes. However, administration of Scl-Ab to estrogen deficient osteocytes is capable of reducing *RANKL*/*OPG* ratio expression.

### Upregulation of CXCL12 an osteoclastogenic regulatory gene in estrogen deficient osteocytes is reversed by Scl-Ab

**Fig. 5:**
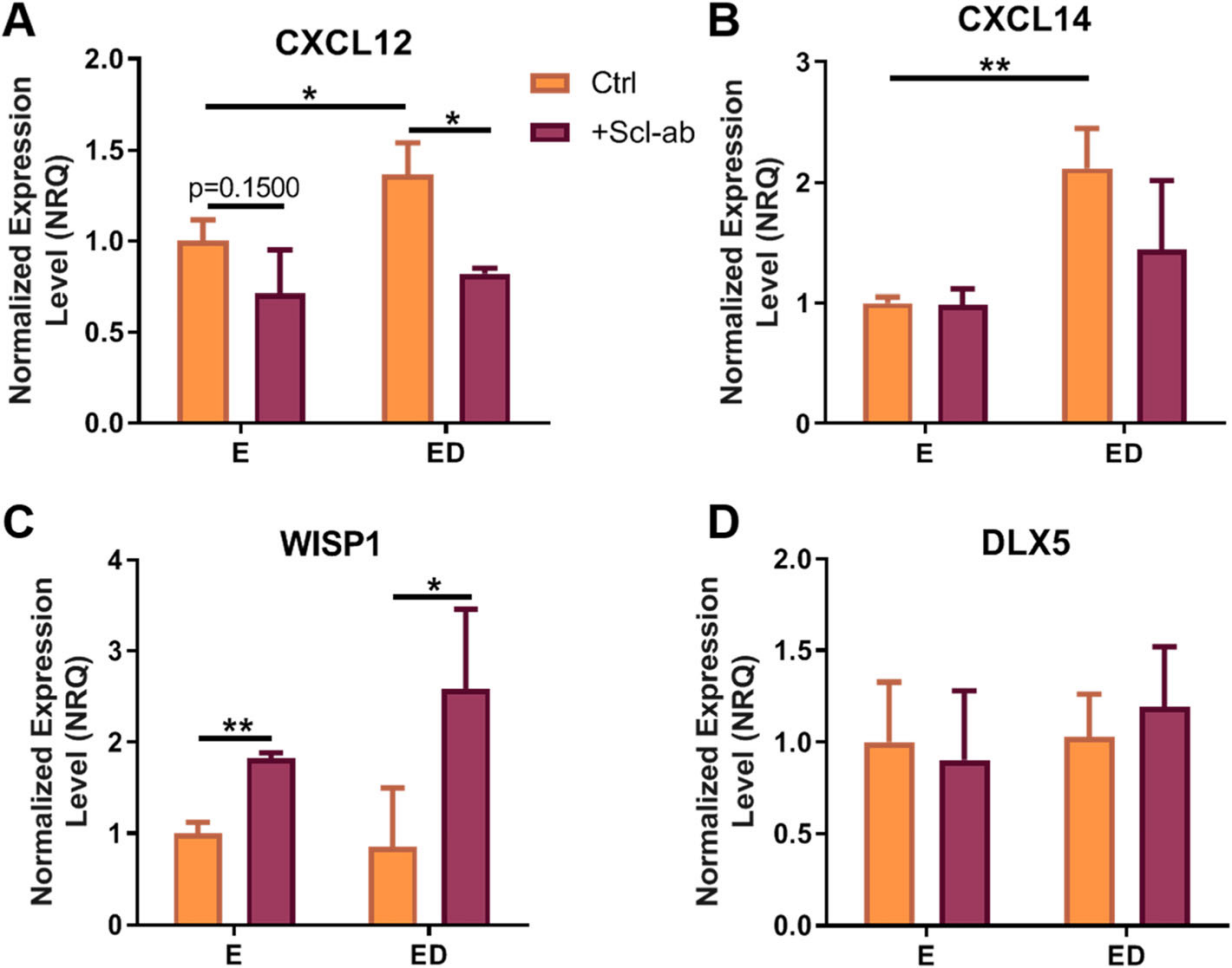
The effect of estrogen deficiency and sclerostin inhibition on osteocytes expression of osteoclastogenic regulatory genes. qRT-PCR analysis of gene expression (relative to estrogen treated group) in OCY454 cells 24 h after mechanical stimulation. **a** CXCL12 expression (*N* = 3, *n* = 9), **b** CXCL14 expression (*N* = 3, *n* = 9), **c** WISP1 expression (*N* = 3, *n* = 9) and **d** DLX5 expression (*N* = 3, *n* = 9). E, estrogen treatment; ED, Estrogen deficient; Scl-Ab, Sclerostin antibody. Student’s t-test.* = *p* < 0.05 and ** = *p* < 0.01

Sclerostin inhibition can regulate expression of osteoclast regulators independent of the RANKL-OPG pathway [43]. For this reason, we analysed the mRNA expression of four secreted osteoclastogenic regulatory genes to determine the effect of estrogen deficiency and sclerostin inhibition on their expression. *CXCL12* and *CXCL14* expression were upregulated in estrogen deficient osteocytes when compared to estrogen treated osteocytes (*p* < 0.05 and *p* < 0.01 respectively) (Fig. 5a, b) but there was no difference in *WISP1* and *DLX5* expression between estrogen deficient and estrogen treated osteocytes (Fig. 5c, d). In estrogen deficient osteocytes, Scl-Ab downregulated *CXCL12* expression (*p* < 0.05) (Fig. 5a). *WISP1* expression was upregulated when estrogen treated and estrogen deficient osteocytes were administered with Scl-Ab, when compared to untreated groups (*p* < 0.01 and *p* = 0.05) (Fig. 5c). However, Scl-Ab had no effect on *DLX5* expression in either estrogen treated or estrogen deficient osteocytes (Fig. 5d). Analysis by two-way ANOVA did not detect a significant interaction between the effects of estrogen and Scl-Ab on the expression of these osteoclastogenic regulatory genes.

### Estrogen deficiency causes downregulation in the Wnt antagonists FRZB, SFRP2 and WIF1 but upregulates SOST expression in osteocytes

**Fig. 6:**
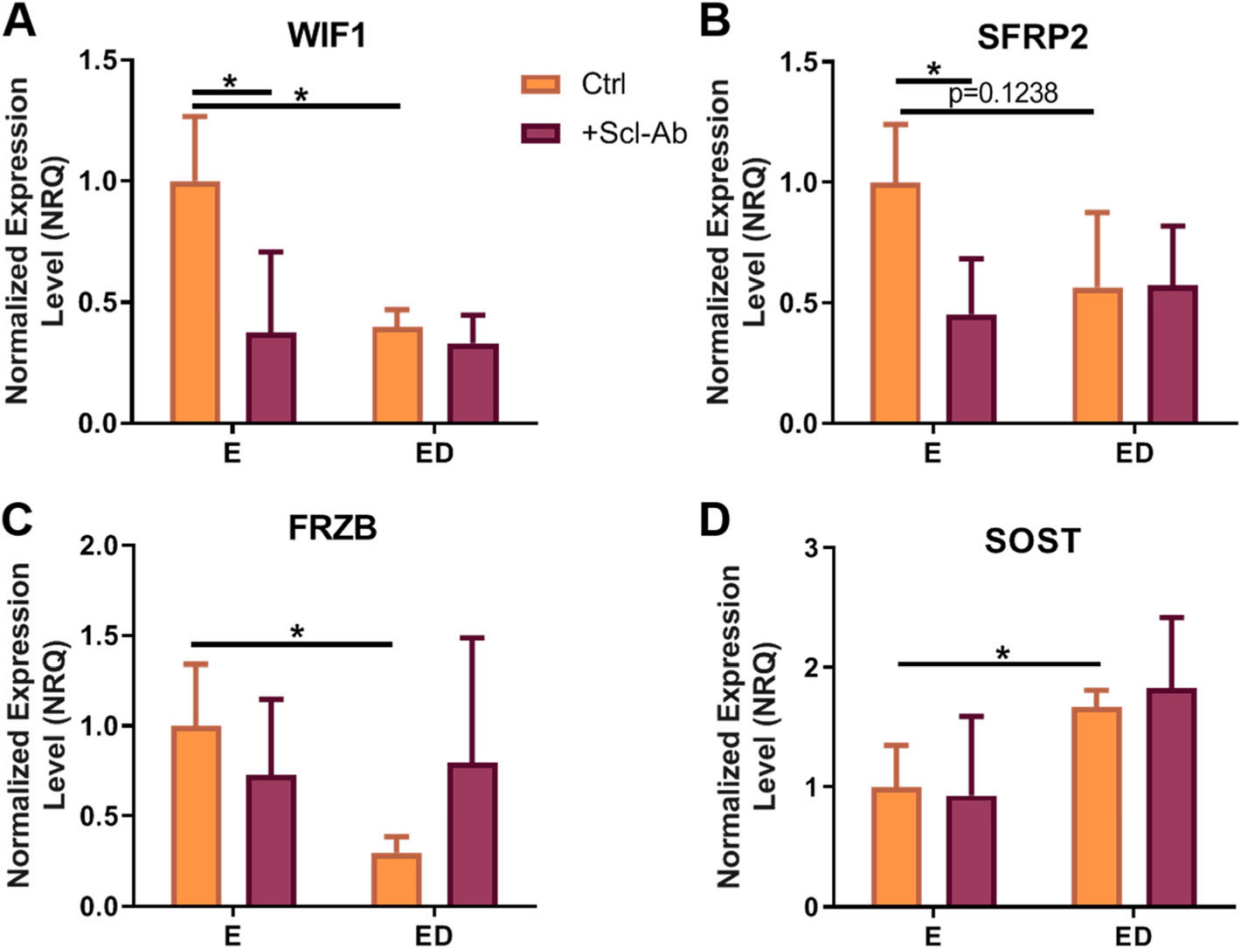
The effect of estrogen deficiency on Wnt antagonist expression. qRT-PCR analysis of gene expression (relative to estrogen treated group) in OCY454 cells 24 h after mechanical stimulation. **a** WIF1 expression (*N* = 3, *n* = 9), **b** SFRP2 expression (*N* = 3, *n* = 9), **c** FRZB expression (*N* = 3, *n* = 9) and (**d**) SOST expression (*N* = 3, *n* = 9). E, estrogen treatment; ED, Estrogen deficient; Scl-Ab, Sclerostin antibody. Student’s t-test. * = *p* < 0.05

The canonical Wnt signalling pathway is an important regulator of bone homeostasis. There are a number secreted Wnt antagonists, which can be categorised as those that bind to Wnt ligands and those that bind to the Wnt co-receptor LRP5/6 [39, 40]. We analysed the mRNA expression of a number of Wnt antagonists including; *WIF1*, those belonging to the soluble frizzled-related protein family; *SFRP2* and *FRZB*, as well the gene that encodes for sclerostin; *SOST*. Under estrogen deficient conditions there was a downregulation in *WIF1* (*p* < 0.05) and *FRZB* (*p* < 0.05) expression compared to estrogen treated osteocytes (Fig. 6a, c). *SOST* expression was upregulated in estrogen deficient osteocytes compared to estrogen treated osteocytes (*p* < 0.05) (Fig. 6d). Administration of Scl-Ab to estrogen-treated osteocytes downregulated expression of *WIF1* (*p* < 0.05) and *SFRP2* (*p* < 0.05) but had no effect on *FRZB* or *SOST* expression compared estrogen-treated osteocytes that received no Scl-Ab (Fig. 6). However, Scl-Ab had no significant effect of Wnt antagonist expression when administered to estrogen deficient osteocytes (Fig. 6). Analysis by two-way ANOVA did not detect a significant interaction between the effects of estrogen and Scl-Ab on the expression of these Wnt antagonists.

## Discussion

In this study, we exposed osteocytes to oscillatory fluid flow to more accurately represent the in vivo environment and reported for the first time that under estrogen deficiency, a significant increase in osteocyte-induced osteoclastogenesis and resorption occurs compared to estrogen-treated groups. Interestingly, we report that administration of Scl-Ab reduces the *RANKL*/*OPG* ratio and upregulates *WISP1* gene expression by estrogen deficient osteocytes and these changes are associated with a reduction in osteoclastogenesis by BMM cells in co-culture. Scl-Ab exerts similar effects on estrogen treated osteocytes, decreasing osteoclastogenesis in primary BMM cells. In addition, bone resorption is reduced, and both *CTSK* and *NFATc1* gene expression are down-regulated in estrogen treated BMM cells following Scl-Ab administration. This study provides an enhanced understanding of the biological changes underpinning decreases in bone resorption following Scl-Ab treatment observed in vivo by revealing that the Scl-Ab can reduce pro-osteoclastogenic cell signalling between osteocytes and osteoclasts.

There are a number of limitations to this study, which must be addressed. Firstly, the osteocyte cell line OCY454 were used rather than primary osteocytes. OCY454 cells are a relatively new cell line developed by Dr. Pajevic (Boston University), which have been shown to secrete significant levels of soluble sclerostin after they are differentiated [51]. They are also mechanosensitive and have been shown to increase their expression of sclerostin in response to microgravity in vitro [51]. Based on these characteristics, OCY454 cells are a promising candidate to study in vitro effects of sclerostin and mechanical stimulation, and their effects on osteocyte-induced osteoclastogenesis, compared to other osteocyte cell lines, such as MLO-Y4 cells, which express very low levels of sclerostin [51]. Secondly, here all osteocytes were cultured in a monolayer and mechanically stimulated using a parallel plate flow chamber to mimic the in vivo environment more accurately, rather than culturing the osteocytes under static conditions, this is an oversimplification of the stresses experienced by osteocytes in vivo*.* However, parallel plate flow chambers have been widely used to study osteocyte mechanobiology as they allow downstream chemical events to be easily studied [26, 27, 35, 52]. In the clinical setting, Scl-Ab is administered systemically, therefore we treated both OCY454 and BMM cells with Scl-Ab in vitro. Deletion of β-catenin in osteoclast precursors has been shown to result in an increase in osteoclast number and enhanced resorption. This could suggests that sclerostin might have a direct role in osteoclast differentiation, which is independent of RANKL and OPG [53], however addition of sclerostin to splenocyte or PBMC monocultures had no effect on osteoclastogenesis, indicating that sclerostin does not directly influence these cells [42]. We report differences in osteocyte-mediated differentiation and response to Scl-Ab in RAW264.7 cells and primary BMM cells. Whilst RAW264.7 cells have been used to study osteoclastogenesis for more than 20 years, due to their ease of culture and wide availability, there are differences in cellular programming compared to BMM cells, which have demonstrated a superior ability to resorb bone [54, 55]. It should also be noted that RAW264.7 cells are derived from adult male BALB/c mice (Abelson leukaemia virus-induced tumor), whereas the BMMs cells were isolated from bone marrow of healthy adult female C57BL/6 mice. Thus sex specific differences may explain the differences observed. Indeed ERα inactivation in female mice resulted in a reduction in load induced bone formation, whereas no effect was observed in male mice [56]. Finally, in vitro stimulation of pre- and post-menopausal conditions by the addition or withdrawal of estrogen results in a sudden decrease in estrogen levels, whereas in humans, serum estradiol levels deplete over a 4 year period [57]. Nonetheless, the results show significant and important difference in osteocyte-induced osteoclastogenesis between the continued estrogen administrated and the estrogen deficient groups and thus provide an advanced understanding of role of estrogen status on osteocyte signalling.

It has previously been reported that estrogen deficient MLO-Y4 cells display an increase in *RANKL*/*OPG* expression [27], and our study supports this as an increase in *RANKL*/*OPG* ratio in OCY454 cells was also seen. Importantly, we report for the first time here that this increase in *RANKL*/*OPG* ratio resulted in increased osteocyte-induced osteoclastogenesis in primary cells (BMM), and *NFATc1* expression was also upregulated, which is a master regulator of osteoclastogenesis [48]. We also report an increase in *CXCL14* and *CXCL12* expression by estrogen deficient osteocytes, which are regulatory genes that promote chemoattractant recruitment of osteoclasts and their precursors [46, 47]. *CXCL12* signalling has also been shown to promote the development and survival of osteoclasts [47]. Therefore, the increases in osteocyte-induced osteoclastogenesis during estrogen deficiency reported here may be explained by changes in *RANKL*, *OPG*, *CXCL12* and *CXCL14* gene expression. Increases in osteocyte-induced osteoclastogenesis in estrogen deficiency occurred through paracrine signalling and through cell-cell contact signalling to similar effects in BMM cells. Although membrane-bound RANKL has been shown to be more potent than soluble RANKL [58], the results presented here suggests that under estrogen deficient conditions this may not be the case. In vitro osteoblast-osteoclast studies previously reported an increase in matrix degradation in estrogen deficient groups compared to estrogen treated group [59]. Here we report a significant increase in bone resorption through use of bovine bone resorption assays, in estrogen deficient groups compared to estrogen treated groups.

Mechanical loading has been shown to downregulate *SOST* expression by TGF-β dependant mechanisms [34, 35, 60, 61]. However, here we report that estrogen deficient osteocytes following loading failed to downregulate *SOST* expression compared to estrogen treated osteocytes. This is contrary to a previous study, which observed a downregulation in *SOST* expression in estrogen deficient osteocytes exposed to oscillatory fluid flow [28]. It should be noted, that the previous study used MLO-Y4 cells, which express extremely low levels of *SOST* compared to the OCY454 cells used in this study [51]. However, the upregulation of *SOST* expression observed here in estrogen deficient OCY454 cells is supported by increases in *SOST* expression seen in human postmenopausal bone and OVX mice [36, 37]. Interestingly, we report that Wnt antagonists *WIF1*, *SFRP2* and *FRZB*, which inhibit Wnt signalling by binding to Wnt ligands, were downregulated in estrogen deficiency. Estrogen receptor alpha (ERα), is involved in bone cells adaptive response to mechanical strain [62–65]. Estrogen receptor signalling has demonstrated to work synergistically with Wnt3A to promote osteogenic differentiation [66]. Additionally, activation of β-catenin and its translocation to the nucleus has been shown to be facilitated by ERα [67]. It has been reported that when estrogen levels are low, or an estrogen receptor antagonist (Fulvestrant) is present, the amount of functional ERα available is reduced, and so there is an insufficient amount of receptors to transduce mechanical strain to biochemical responses [68, 69]. This has been shown to result in reduced accumulation of β-catenin and therefore a reduction in the transcription of Wnt target genes, leading to a decrease in bone formation [67]. We propose that the decrease in Wnt antagonists (*WIF1*, *SFRP2* and *FRZB*) act to compensate for the alteration in mechanotransduction seen in estrogen deficiency [67], to promote Wnt signalling and therefore increase activation β-catenin leading to increased osteogenic responses. Supporting this, MC3T3-E1 cells have been reported to upregulate *SFRP1* following mechanical loading [70]. Future studies should investigate whether this response in osteoblastic cells is abrogated in estrogen deficient conditions, similar to what we reported here in osteocytes. Scl-Ab administration down-regulated both *WIF1* and *SFRP2* expression in estrogen-treated cells, however it appeared to have no significant influence on estrogen deficient cells. This may be a result of our experimental timelines, in which we analysed WNT antagonist expression after only 2 days of Scl-Ab treatment, whereas an in vivo study showed an upregulation in Wnt antagonist expression after 1 week of Scl-Ab administration [71].

Antibodies against sclerostin have demonstrated the ability to promote bone formation leading to increase bone mass and bone strength in both animal studies and clinical trials [4, 8–11]. Interestingly Scl-Ab has shown to decrease *RANKL*/*OPG* ratio, osteoclastogenesis in ex vivo cultures from spinal cord injury rat models that undergo bone loss [72]. We report similar effects in terms of osteoclastogenesis, whereby Scl-Ab administration to estrogen deficient osteocytes resulted in a decrease in osteoclastogenesis and resorption, as determined by TRAP staining, TRAP activity, resorption assays and *NFATc1* and *CTSK* expression. This was at least in part due to a decrease in *RANKL*/*OPG* expression in estrogen deficient osteocytes treated with Scl-Ab. A microarray study of osteocyte-enriched trabecular bone from Scl-Ab treated rats, revealed a decrease in *RANKL*/*OPG* ratio in osteocytes from Scl-Ab treated rats [73]. In addition to this, studies have reported that the Scl-Ab upregulated *WISP1* expression in osteocytes, a known negative regulator of osteoclastogenesis [43, 45, 73]. In this study we also report that Scl-Ab administration results in an upregulation of *WISP1* expression by both estrogen treated and estrogen deficient osteocytes. Furthermore, *CXCL12* expression was downregulated in estrogen deficient osteocytes that received the Scl-Ab, which may decrease the recruitment, development and survival of osteoclasts [46, 47]. Thus a decrease in *RANKL*/*OPG* ratio and *CXCL12* expression along with an increase in *WISP1* expression following treatment with the Scl-Ab might explain the decrease in osteoclastogenesis and resorption reported here. Scl-Ab administration was shown to exert similar effects on estrogen treated osteocytes, reducing *RANKL*/*OPG* expression and inhibiting osteocyte-induced bone resorption. Analysis of the data (by two-way ANOVA) revealed a significant interaction between estrogen and Scl-Ab administration in terms of the bone resorption, *RANKL* and *OPG* expression data; thus it is proposed that estrogen and Scl-Ab synergistically inhibit *RANKL*/*OPG* expression and bone resorption. There was no significant interaction between the effects of estrogen and Scl-Ab in terms of TRAP staining, TRAP activity, osteoclastogenic regulatory genes (*WISP1*, *CXCL12*, *CXCL14*, and *DLX5*) and Wnt antagonists.

## Conclusion

We report that under postmenopausal conditions, an increase in *RANKL*/*OPG* ratio is expressed by osteocytes compared to estrogen-treated osteocytes and a significant increase in osteocyte-induced osteoclast formation occurs leading to increased bone resorption. We demonstrate that in estrogen deficient conditions there is an upregulation of *SOST* expression but a downregulation in other Wnt antagonists e.g. *WIF1*, *FRZB* following mechanical loading, which may indicate a compensatory mechanism for alteration in mechanosensation and mechanotransduction seen in osteocytes. We propose that sclerostin plays an important role in the enhanced pro-osteoclastogenic signalling between osteocyte-osteoclast in estrogen deficient conditions. We are the first to show that administration of Scl-Ab reduces pro-osteoclastogenic signalling between osteocytes and osteoclasts, which leads to reduced bone resorption. This study provides enhanced understanding of the biological changes underpinning reduction in bone resorption seen in animal and clinical studies following Scl-Ab treatment and forms part of the wider mechanism of action of Scl-Ab, as potent bone-forming agents in severely osteoporotic patients who need rapid increases in bone mineral density to prevent fragility fracture.

## Methods

### Cell culture

Murine OCY454 osteocyte cells were purchased from the Centre for Skeletal Research Bone Cell Core, an NIH-funded program (P30AR075042), which is supported by NIAMS. OCY454 cells were expanded on type I collagen (0.15 mg/ml in 0.02 M acetic acid) coated T-175 flasks in α-MEM supplemented with 1% L-glutamine, 2% antibiotics, and 10% FBS. OCY454 cells were routinely cultured at the permissive temperature of 33 °C, cells were then trypsinized and placed in non-collagen coated T-175 flasks. After 3 days, cells were differentiated by being transferred to the semi-permissive temperature of 37 °C for 15 days [51] before being treated with 17β-estradiol, cells were maintained in a humidified environment at 5% CO2.

Osteoclast differentiate from cells of the monocyte/macrophage lineage [19], based on this bone marrow macrophages (BMM) were isolated from 8 month old female C57BL/6 mice based on [74]. All animal work was carried out under license from the Animal Care and Research Ethics Committee (ACREC) of the National University of Ireland Galway and the Health Products Regulatory Authority (HPRA), the national authority for scientific animal protection in Ireland. Briefly, hind limbs were removed of muscle and tissue, each end of the bone was sectioned, bone marrow aspirate was centrifuged and plated at 5 × 10^6^ cells in 10 cm bacteriological petri dishes. The bone marrow macrophages were differentiated in the presence of α-MEM supplemented with 1% L-glutamine, 2% antibiotics, 10% FBS and 20% L929 fibroblast cell conditioned media (source of M-CSF). Once macrophages were confluent (approximately 1 week after isolation), cells were trypsinized and scraped, and frozen down or used straight away. BMM cells were used at passage 3 or below. Additionally, RAW264.7 cells were purchased from the American Type Culture Collection (ATCC, Manassas, VA, USA), to assess whether the commonly used male derived cell line responded similarly to primary BMM cells which were isolated from female mice. RAW264.7 cells were expanded in Dulbecco’s modified Eagle’s medium with 1% antibiotics, 1% L-Glutamine, and 10% heat-inactivated FBS (HyClone). BMM and RAW264.7 cells were cultured in a humidified atmosphere at 37 °C in 5% CO_2_.

### Estrogen treatment regimes

The effect of supplementing culture media with pre-menopausal levels of estrogen (E) on OCY454 osteocyte behaviour under oscillatory flow conditions in vitro was investigated. Following 15 days of expansion in α-MEM culture media at 37 °C, OCY454 cells were treated with estrogen (E: 10 nM 17β-estradiol) for 6 days. To simulate postmenopausal conditions (ED), we implemented a model that first accustomed osteocytes to estrogen before a subsequent period where estrogen supplementation was discontinued, known as estrogen withdrawal, based on our previous established postmenopausal model using MC3T3-E1 and MLO-Y4 cells [27, 59]. OCY454 cells were treated with 17β-estradiol (10 nM) for 3 days before it was withdrawn from the media for a further 3 days. In total OCY454 cells were culture at differentiated 37 °C for 21 days before being exposed to oscillatory fluid flow.

### Sclerostin inhibition

Sclerostin antibody (Scl-Ab VI) was kindly provided by UCB pharma (UCB pharma, UK/Amgen Inc. USA) and was stored at 5 mg/ml aliquots at − 80 °C. In order to obtain sufficient neutralisation of sclerostin, we treated with ≥30x higher concentration than the amount of sclerostin produced by OCY454 cells. A previous study reported that OCY454 cells cultured at 37 °C for 14 days produce approximately 75 pg/ml of sclerostin [51], based on this, a non-cytotoxic concentration of 300 ng/ml was chosen to ensure sufficient sclerostin neutralisation. Untreated groups received PBS as a vehicle, similar to in vivo Scl-Ab studies [9–11, 71, 75].

### Mechanical stimulation

Osteocytes are the main mechanosensors in bone and are subjected to various forms of mechanical loading [15, 76, 77]. Therefore to capture the in vivo environment more accurately, all osteocytes were subjected to oscillatory fluid flow. Prior to mechanical stimulation OCY454 cells from each of the treatment groups (E and ED) were seeded at 200,000 cells per collagen coated glass slide (76 mm × 26 mm) (collagen coating was used to prevent cell detachment during fluid flow) and were cultured for a further day either under (a) continued estrogen (E) or (b) estrogen deficient conditions (ED). A subset of these groups, were also treated with 300 ng/ml Scl-Ab, 24 h before stimulation. Laminar oscillatory fluid flow was applied to OCY454 cells using a parallel plate system, which comprised of a syringe pump (NE-1600, New Era Pump Systems, Farmingdale, NY, USA), parallel plate chambers and individual media reservoirs connected through gas-permeable silicone tubing (Cole-Parmer, Vernon Hills, IL, USA) [52]. The OFF loading regime subjected the OCY454 cells to a shear stress of 1 Pa at 0.5 Hz for 1 h, which is within the range of shear stresses experiences by osteocytes in vivo [76, 78–80]. Before and after oscillatory fluid flow, slides were rinsed with PBS (× 3) to remove residual media, to ensure that conditioned media collected did not contain 17β-estradiol. After flow, fresh media (without estrogen supplementation) was applied and cells were then cultured for 24 h before conditioned media was collected for further experiments (described below). Cells treated with Scl-Ab 24 h prior to flow continued to be treated with 300 ng/ml Scl-Ab (i.e. when CM was collected OCY454 cells had been exposed to 300 ng/ml Scl-Ab for 48 h).

### Conditioned media experiments

Conditioned media from all cell treatment groups (E, E+ Scl-Ab, ED and ED-Scl-Ab) was centrifuged at 1500 rpm and then frozen at − 80 °C in 1 mL aliquots. RAW264.7 cells and BMM were seeded at 5000 and 12,000 cells respectively per well in a 96 well plate and then treated with 50% conditioned media and 50% expansion media (DMEM). Cells were then cultured in the presence of 15 ng/mL of RANKL for 5 days.

### Bone resorption assessment

6 mm bone discs with a thickness of between 0.4–0.6 mm were created from bovine metatarsals based on [81]. Bone discs were soaked in 70% ethanol and placed in ultrasonic bath (VWR, Dublin, Ireland) at room temperature for 15 min. Prior to culture, discs were sterilised under UV light, BMM cells were seeded on discs at 14,000 cells per discs in a 96 well plate. Cells were cultured with 50% CM and 15 ng/ml RANKL for 10 days, on day 7 concentrated hydrochloric acid (HCL) was added to the culture media to achieve pH 6.9 to induce a more acidic environment necessary for osteoclast resorption [81]. On day 10 the experiment was terminated and cells were removed by sonication in 0.25 M ammonium hydroxide for 5 min. Resorption pits were stained using 1% Toluidine blue in 1% sodium borate solution. Images were acquired using a light microscope (Olympus BX43, Olympus, Tokyo, Japan) and quantified using ImageJ software, in which images were colour-thresholded and percentage area resorbed was quantified.

### Tartrate-resistant acid phosphatase (TRAP) staining

At the end of the co-culture and conditioned media experiments RAW 264.7 cells and BMMs were rinsed with PBS and fixed with 4% Paraformaldehyde. They were then rinsed with PBS and stained for tartrate-resistant acid phosphatase (TRAP) activity with a commercial kit and counterstained with Gill No. 3 hematoxylin for 3 min. Staining was also performed on RAW264.7 and BMM cells, which received expansion media only (negative control) and cells that received expansion media with only supplementation of 15 ng/mL RANKL (positive control). Images were acquired using a light microscope (Leica DMi1, Leica Biosystems, Wetzlar, Germany) and quantified using ImageJ software, in which images were colour thresholded and the percentage area covered by osteoclasts was quantified. TRAP-positive cells with 3 or more nuclei were considered to be osteoclasts.

### TRAP activity assay

At the end of the conditioned media and co-culture experiments, culture media was collected from wells and secreted TRAP activity was detected. The TRAP activity assay involved measuring enzyme activity by the conversion of p-nitrophenylphosphate (20 nM) to p-nitrophenol in the presence of 80 mM sodium tartrate and was expressed as optical density at 405 nm using a microplate reader (Synergy HT, Biotek Instruments Inc., Winooski, VT, USA).

### Co-culture experiments

Mechanically stimulated OCY454 cells from each group (E, E+ Scl-Ab, ED and ED + Scl-Ab) were seeded at 25,000 cells per well in a 48 well plate. After 24 h RAW264.7 cells were seeded on top of OCY454 cells at 10,000 per well based on a previous study [59] and BMM cells were seeded at 34,000 cells per well. Cells were co-cultured in the presence of 15 ng/mL of RANKL for a further 6 days. To mimic systemic administration in humans, Scl-Ab treatment groups continued to receive 300 ng/ml Scl-Ab throughout the 6 days of co-culture (i.e. both osteocytes and osteoclast precursors were cultured in the presence of Scl-Ab).

### qRT-PCR

BMM cells were exposed to 50% CM, as described above, and cultured for 5 days. At day 5 mRNA was isolated using High pure RNA isolation kit (Roche Applied Science, Mannheim, Germany). RNA concentration and quality was assessed with a Spectrophotometer/Fluorometer (DS-11 FX, DeNovix, Wilmington, DE, USA)). cDNA was generated using the QuantiNova Reverse Transcription Kit. qRT-PCR was performed on resultant cDNA template on a StepOne plus real-time PCR machine (Applied Biosystems, Foster City, CA, USA) using a QuantiNova SYBR Green PCR Kit and custom designed primers (IDT, Coralville, IA, USA) for Nuclear factor of activated T-Cells cytoplasmic 1 (NFAT*c1*), and Cathepsin K (*CTSK*) (Primer sequences are shown in Supplementary Table 1).

mRNA from OCY454 cells was isolated 24 h after exposure to oscillatory fluid flow and transcribed to cDNA using the same protocol as described above. Expression of the following genes were assessed; Receptor activator of nuclear factor kappa-Β ligand (*RANKL*), Osteoprotegerin (*OPG*), Wnt inhibitory factor 1 (*WIF1*), Frizzled-related protein (*FRZB*), Secreted frizzled related protein 2 (*SFRP2*), Sclerostin (*SOST*), Wnt1-inducible signalling pathway protein-1 (*WISP1*), C-X-C motif chemokine ligand 12 (*CXCL12*), C-X-C motif chemokine ligand 14 (*CXCL14*) and Distal-less homeobox 5 (*DLX5*) (Primer sequences are shown in Supplementary Table 1). The normalised relative quantities (NRQ) of each sample (BMM and OCY454 samples) were calculated with reference to ribosomal protein large subunit P0 (*RPLP0*) (Reference gene was identified through qbase+ software). Data was analysed using the Pfaffl method [82].

### Statistical analysis

Data are representative of 3 independent experiments performed in triplicate and are presented as mean ± SD. Statistical analysis was performed by unpaired two-tailed Student’s t-test to examine the effect of estrogen treated groups vs. estrogen deficient groups or Scl-Ab treated group vs. untreated groups. In addition, the interaction between the effects of estrogen and Scl-Ab was also tested though two-way ANOVA analysis. A value of *p* < 0.05 was regarded as statistically significant. Technical replicates are represented as N, while biological replicates are represented as n.

## Supplementary Information

**Additional file 1: Supplementary Figure 1**. The effect of CM from OCY454 cells on osteoclastogenesis in RAW264.7 cell cultures. Images show (A) multinucleated TRAP+ osteoclasts (orange arrows) (*N* = 3, *n* = 9) after RAW264.7 cells were treated with CM from OCY454 cells. Quantification of images showing (B) percentage surface area covered by TRAP+ multinucleated cells (*N* = 3, *n* = 9), (C) TRAP activity in supernatant collected from multinucleated cells (*N* = 3, *n* = 9). E, estrogen treatment; ED, Estrogen deficient; Scl-Ab, Sclerostin antibody. Student’s t-test. * = *p* < 0.05, ** = *p* < 0.01 and **** = *p* < 0.0001. **Supplementary Figure 2**. The effect of sclerostin inhibition on osteocyte induced osteoclastogenesis in a co-culture system. (A) Images show TRAP+ osteoclasts (orange arrows) formed when RAW264.7 cells were co-cultured with OCY454 cells for 6 days. (*N* = 3, *n* = 9). Quantifications of images showing (B) percentage area covered by the TRAP+ cells (*N* = 3, *n* = 9) and (C) TRAP activity in supernatant collected from OCY454-RAW264.7 co-cultures after 6 days (*N* = 3, *n* = 9). E, estrogen treatment; ED, Estrogen deficient; Scl-Ab, Sclerostin antibody. Student’s t-test. * = *p* < 0.05, *** = *p* < 0.001 and **** = *p* < 0.0001. **Supplementary Table 1**. Primer sequences used in qRT-PCR.

## Data Availability

The datasets generated during and/or analysed during the current study are available from the corresponding author on reasonable request.

## Abbreviations

TRAP: Tartrate-resistant acid phosphatase
*CTSK*: Cathepsin K
*NFATc1*: Nuclear factor of activated T-cells, cytoplasmic 1
BMM: Bone marrow macrophage
Scl-Ab: Sclerostin antibody
LRP 5/6: Low-density lipoprotein receptor-related protein 5/6
*OPG*: Osteoprotegerin
*WIF1*: Wnt inhibitory factor 1
*FRZB*: Frizzled-related protein
*SFRP2*: Secreted frizzled related protein 2
*SOST*: Sclerostin
*WISP1*: Wnt1-inducible signalling pathway protein-1
*CXCL12*: C-X-C motif chemokine ligand 12
*CXCL14*: C-X-C motif chemokine ligand 14
*DLX5*: Distal-less homeobox 5
*RANKL*: Receptor activator of nuclear factor kappaB ligand
E: Estrogen
ED: Estrogen Deficient
OFF: Oscillatory fluid flow
NRQ: Normalised relative quantities
α-MEM: α-minimum essential medium
DMEM: Dulbecco’s modified eagle media
