# Supplementary material for: Scl-Ab reverts pro-osteoclastogenic signalling and resorption in estrogen deficient osteocytes
Source: BMC Mol Cell Biol. 2020 Nov 4;21:78. doi: 10.1186/s12860-020-00322-w (PMC7643443; doi:10.1186/s12860-020-00322-w)

**Supplementary figures**


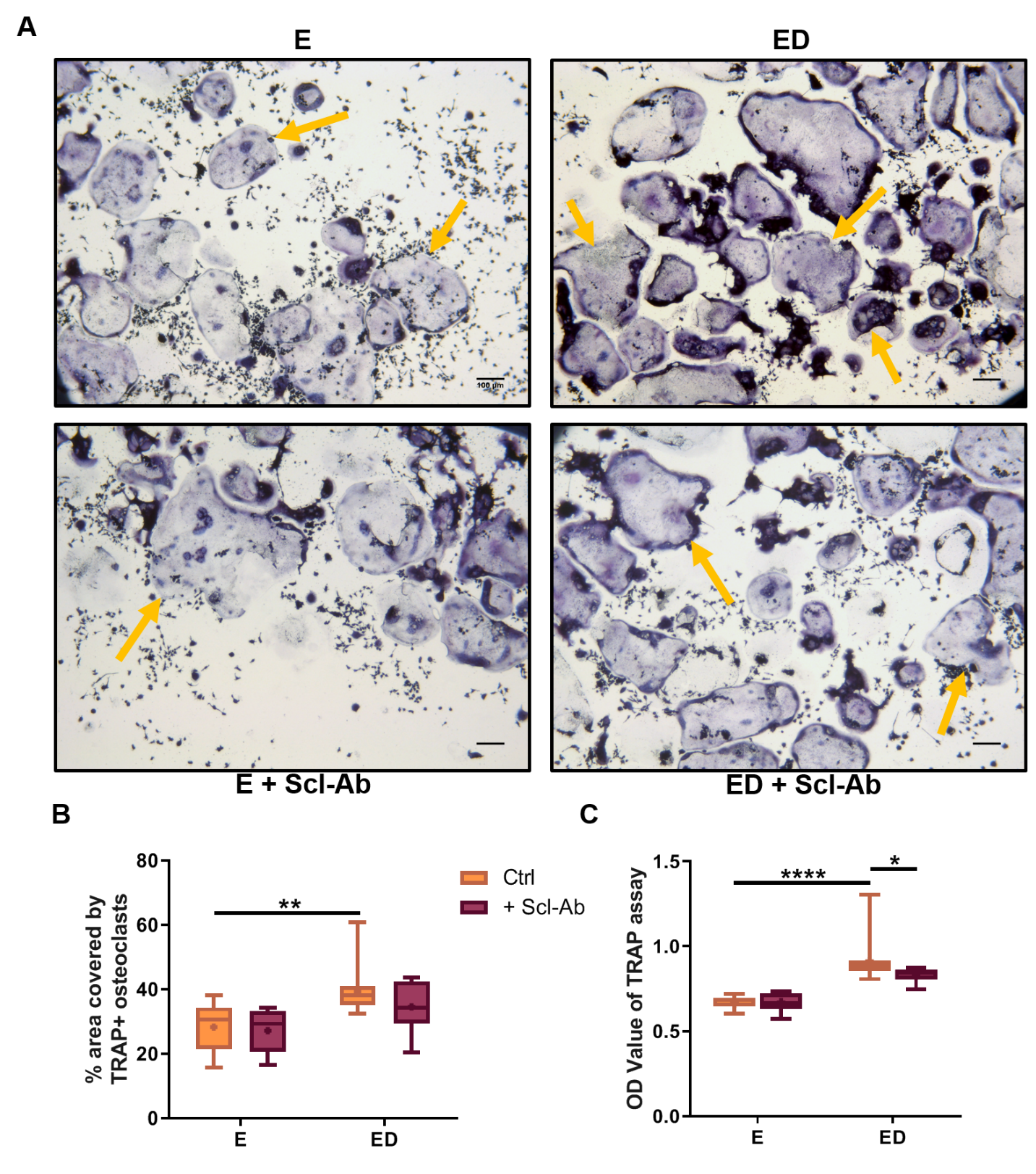


Supplementary Figure 1: The effect of CM from OCY454 cells on osteoclastogenesis in RAW264.7 cell cultures. Images show (A) multinucleated TRAP+ osteoclasts (orange arrows) (N=3, n=9) after RAW264.7 cells were treated with CM from OCY454 cells. Quantification of images showing (B) percentage surface area covered by TRAP+ multinucleated cells (N=3, n=9), (C) TRAP activity in supernatant collected from multinucleated cells (N=3, n=9). E, estrogen treatment; ED, Estrogen deficient; Scl-Ab, Sclerostin antibody. Student’s t-test. *=p<0.05, **=p<0.01 and ****=p<0.0001


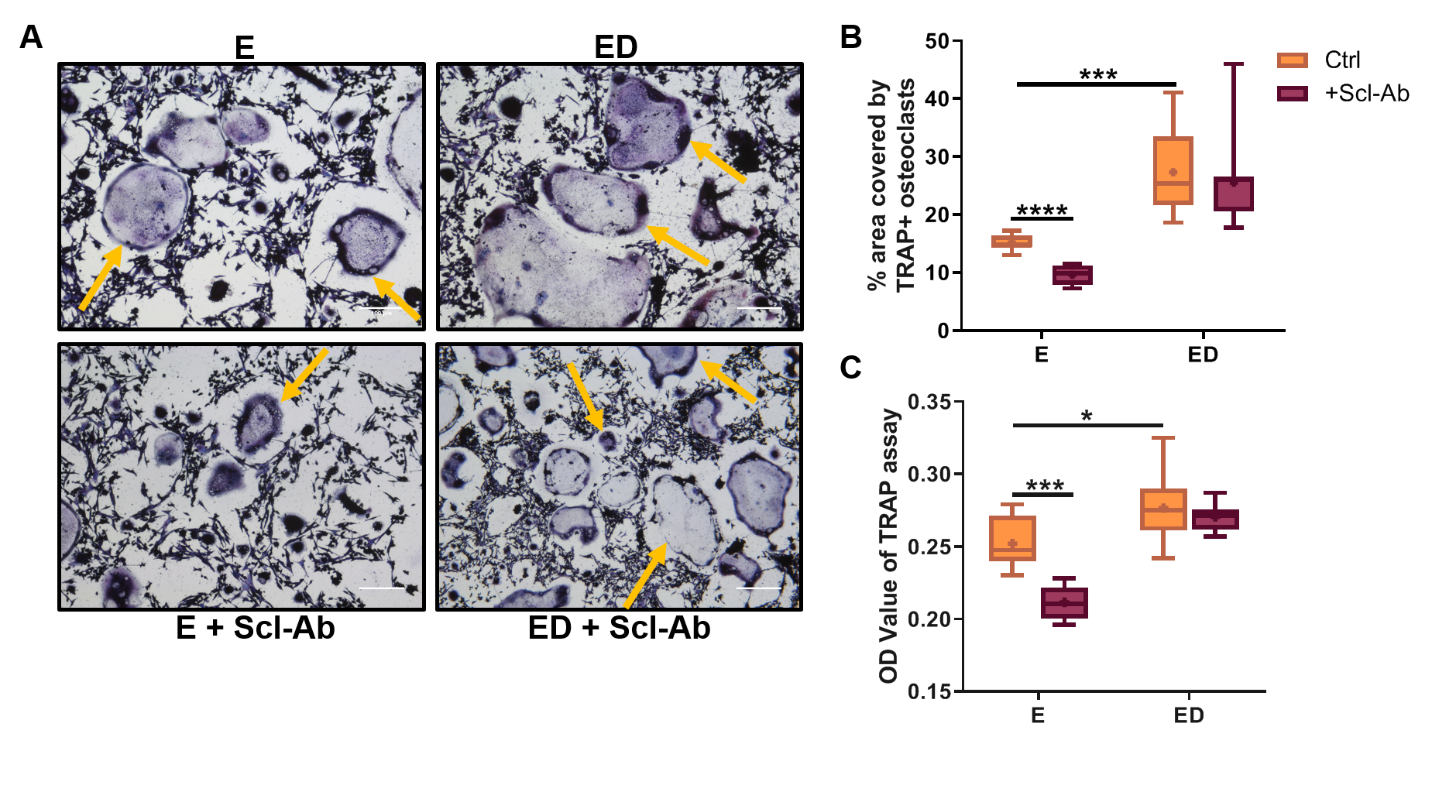


Supplementary Figure 2: The effect of sclerostin inhibition on osteocyte induced osteoclastogenesis in a co-culture system. (A) Images show TRAP+ osteoclasts (orange arrows) formed when RAW264.7 cells were co-cultured with OCY454 cells for 6 days. (N=3, n=9). Quantifications of images showing (B) percentage area covered by the TRAP+ cells (N=3, n=9) and (C) TRAP activity in supernatant collected from OCY454-RAW264.7 co-cultures after 6 days (N=3, n=9). E, estrogen treatment; ED, Estrogen deficient; Scl-Ab, Sclerostin antibody. Student’s t-test. *=p<0.05, ***=p<0.001 and ****=p<0.0001

Supplementary table 1: Primer sequences used in qRT-PCR


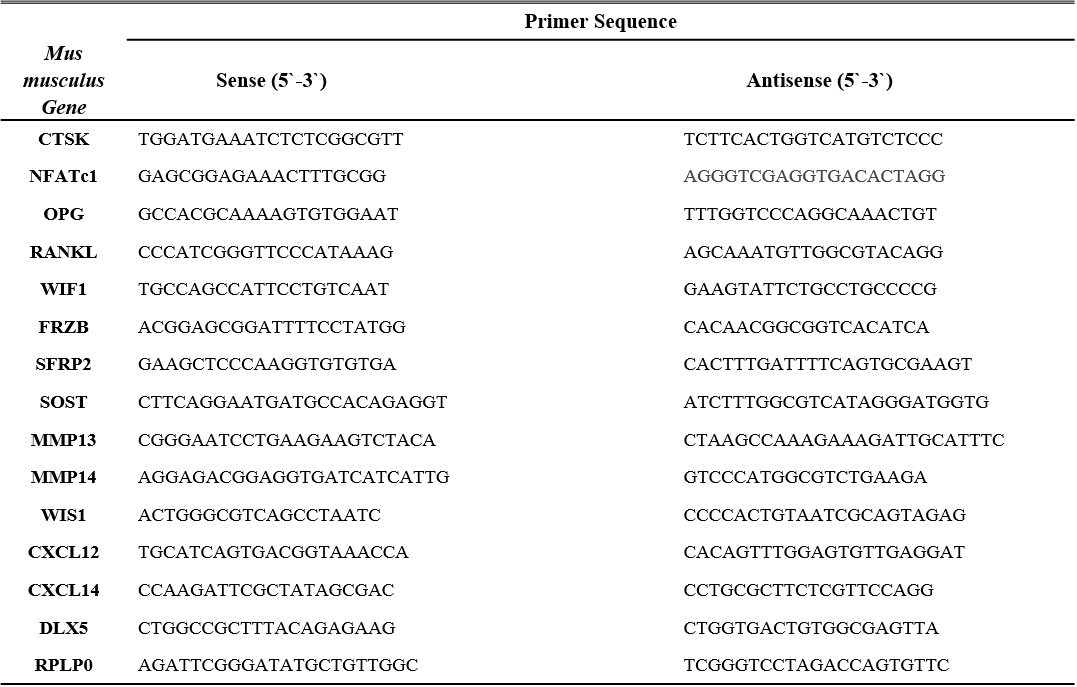

Supplement: Supplementary file 1 — Additional file 1: Supplementary Figure 1. The effect of CM from OCY454 cells on osteoclastogenesis in RAW264.7 cell cultures. Images show (A) multinucleated TRAP+ osteoclasts (orange arrows) (N = 3, n = 9) after RAW264.7 cells were treated with CM from OCY454 cells. Quantification of images showing (B) percentage surface area covered by TRAP+ multinucleated cells (N = 3, n = 9), (C) TRAP activity in supernatant collected from multinucleated cells (N = 3, n = 9). E, estrogen treatment; ED, Estrogen deficient; Scl-Ab, Sclerostin antibody. Student’s t-test. * = p < 0.05, ** = p < 0.01 and **** = p < 0.0001. Supplementary Figure 2. The effect of sclerostin inhibition on osteocyte induced osteoclastogenesis in a co-culture system. (A) Images show TRAP+ osteoclasts (orange arrows) formed when RAW264.7 cells were co-cultured with OCY454 cells for 6 days. (N = 3, n = 9). Quantifications of images showing (B) percentage area covered by the TRAP+ cells (N = 3, n = 9) and (C) TRAP activity in supernatant collected from OCY454-RAW264.7 co-cultures after 6 days (N = 3, n = 9). E, estrogen treatment; ED, Estrogen deficient; Scl-Ab, Sclerostin antibody. Student’s t-test. * = p < 0.05, *** = p < 0.001 and **** = p < 0.0001. Supplementary Table 1. Primer sequences used in qRT-PCR. [file 12860_2020_322_MOESM1_ESM.docx]
